# Sensitivity of Chaos Measures in Detecting Stress in the Focusing Control Mechanism of the Short-Sighted Eye

**DOI:** 10.1007/s11538-017-0310-5

**Published:** 2017-06-21

**Authors:** Karen M. Hampson, Matthew P. Cufflin, Edward A. H. Mallen

**Affiliations:** School of Optometry and Vision Science, University of Bradford, Bradford, BD7 1DP UK

**Keywords:** Accommodation fluctuations, Chaos theory, Power spectrum, Refractive error

## Abstract

When fixating on a stationary object, the power of the eye’s lens fluctuates. Studies have suggested that changes in these so-called microfluctuations in accommodation may be a factor in the onset and progression of short-sightedness. Like many physiological signals, the fluctuations in the power of the lens exhibit chaotic behaviour. A breakdown or reduction in chaos in physiological systems indicates stress to the system or pathology. The purpose of this study was to determine whether the chaos in fluctuations of the power of the lens changes with refractive error, i.e. how short-sighted a subject is, and/or accommodative demand, i.e. the effective distance of the object that is being viewed. Six emmetropes (EMMs, non-short-sighted), six early-onset myopes (EOMs, onset of short-sightedness before the age of 15), and six late-onset myopes (LOMs, onset of short-sightedness after the age of 15) took part in the study. Accommodative microfluctuations were measured at 22 Hz using an SRW-5000 autorefractor at accommodative demands of 1 D (dioptres), 2 D, and 3 D. Chaos theory analysis was used to determine the embedding lag, embedding dimension, limit of predictability, and Lyapunov exponent. Topological transitivity was also tested for. For comparison, the power spectrum and standard deviation were calculated for each time record. The EMMs had a statistically significant higher Lyapunov exponent than the LOMs ($0.64\pm 0.33$ vs. $0.39\pm 0.20~\hbox {D}/\hbox {s}$) and a lower embedding dimension than the LOMs ($3.28\pm 0.46$ vs. $3.67\pm 0.49$). There was insufficient evidence (non-significant *p* value) of a difference between EOMs and EMMs or EOMs and LOMs. The majority of time records were topologically transitive. There was insufficient evidence of accommodative demand having an effect. Power spectrum analysis and assessment of the standard deviation of the fluctuations failed to discern differences based on refractive error. Chaos differences in accommodation microfluctuations indicate that the control system for LOMs is under stress in comparison to EMMs. Chaos theory analysis is a more sensitive marker of changes in accommodation microfluctuations than traditional analysis methods.

## Introduction

Accommodation is the change in the effective power of the eye’s lens to bring an object into focus on the retina. It is well known that even when fixating on a stationary object of interest, the eye exhibits microfluctuations in accommodation. These fluctuations are a few tenths of a dioptre in magnitude and change at a rate of several Hertz (Charman and Heron 1988, 2015). Since their first observation by Collins (1937), many investigations have been carried out to determine what role, if any, they play in accommodation control. It has been found that the magnitude of the fluctuations is correlated with the objective depth of focus (Yao et al. 2010), and that when the depth of focus is increased, such as by a decrease in pupil size, there is a concomitant increase in the magnitude of the fluctuations (Yao et al. 2010; Gray et al. 1993a, b; Stark and Atchison 1997; Niwa and Tokoro 1998; Gambra et al. 2009). This increase is often attributed to an increase in the magnitude of the low-frequency component (LFC) (${<}0.6~\hbox {Hz}$). Owing to such findings, it has been proposed that the accommodative system monitors the resulting fluctuations in image contrast to help the eye stay in focus (Winn 2000). However, there is still debate over the exact neurological control mechanism. Understanding the nature of the control mechanism is of importance in myopia (short-sightedness) research where changes in the accommodation system, such as in the variability of the accommodative microfluctuations, have been implicated as a factor in myopia onset and progression (Langaas et al. 2008; Langaas and Riddell 2012).

It has been found that accommodative microfluctuations are chaotic in nature (Hampson and Mallen 2012; Sumida et al. 1994). Hence, chaos is a potential factor in the accommodation control mechanism. Despite the traditional meaning of the word chaos, chaotic systems are described completely by underlying laws (Williams 1997). A marker of chaos is sensitivity to initial conditions, the so-called *butterfly effect*. This effectively means that if there is a miniscule change in the initial conditions, the resulting evolution of the system over time will be very different. Chaos exists everywhere in nature, from the dynamics of the weather to the heartbeat. Chaos is advantageous for physiological systems as it allows for complex output behaviour using a low number of input variables (Skinner 1994). Several studies have found that the breakdown or reduction in chaos in physiological signals, such as the heartbeat, is indicative of stress to the system and pathology (Poon and Merrill 1997; Su et al. 2008; Yeragani et al. 2002; Rao and Yeragani 2001). Furthermore, chaos theory analysis has been found to be a more sensitive marker of changes in the dynamics of physiological signals than methods such as power spectrum analysis (Yeragani et al. 2002; Hampson et al. 2013), which is commonly used to analyse the accommodative microfluctuations (Monticone and Menozzi 2011). For example, Hampson et al. (2013) used adaptive optics to correct for fluctuations in visual blur owing to defocus fluctuations. They found that this correction resulted in a decrease in chaos in the accommodation microfluctuations. Changes were not detected when analysing the power spectra of the accommodation signals, however.

The aim of this experiment was to determine whether refractive error and/or accommodative demand affects the level of chaos in microfluctuations in accommodation. The data were also tested for topological transitivity, which is another marker of chaos. The power spectra and standard deviation of the microfluctuations were also determined for comparison.

## Method

### Subjects

Eighteen subjects took part in the study and were classified by their mean spherical equivalent refractive error (SER, sphere + 1/2 cylinder) and age of onset of myopia. The cut-off age separating early-onset versus late-onset myopes was 15 years (McBrien and Millodot 1986). Six emmetropes (EMMs, mean SER $+0.30\pm 0.16~\hbox {D}$), six early-onset myopes (EOMs, mean SER $-4.71 \pm 1.58~\hbox {D}$), and six late-onset myopes (LOMs, mean SER $-1.79\pm 0.75~\hbox {D}$) were recruited from the student cohort of the School of Optometry and Vision Science, University of Bradford. Subjects gave informed consent, and the study was conducted in accordance with the Declaration of Helsinki. Ethical approval was granted by the Institutional Ethical Committee. The median age of each refractive group was 23, 21.5, and 27 years for the EMMs, EOMs, and LOMs, respectively. All subjects were free from ocular pathology and required cylindrical refractive error corrections of ${\le }0.50~\hbox {D}$. All myopic observers were habitual contact lens wearers and wore their ultra-thin spherical soft contact lens correction throughout the experiment.

### Instrumentation

**Fig. 1:**
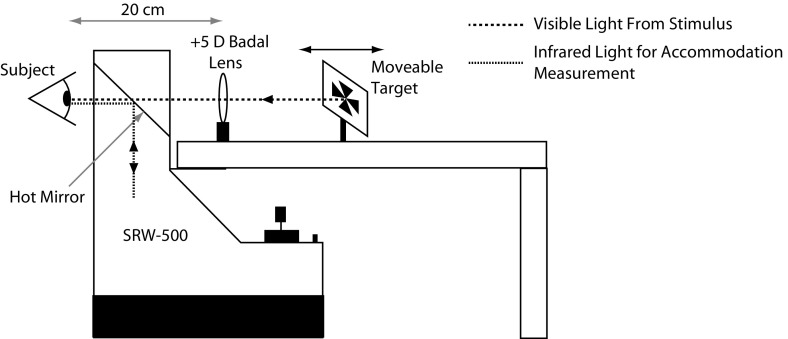
Experimental set-up. The target is viewed via a hot mirror which transmits visible light. The infrared light used for measuring the accommodation fluctuations is reflected into the eye via the hot mirror

Accommodation microfluctuations were measured using the SRW-5000 autorefractor (Grand Seiko Co. Ltd, Hiroshima, Japan) which was modified to allow for continuous recording of accommodation at a sampling rate of 22 Hz, whilst retaining the ability to measure static refractive error (Wolffsohn et al. 2001). The experimental set-up is shown in Fig. 1. The fixation target was a high contrast (90%), black and white Maltese cross, subtending $1.5^{\circ }$ visual angle at the eye and viewed via a $+$5.00 D Badal lens system. The target was viewed in open view via a hot mirror that is part of the instrument. This mirror transmits visible light from the target. The infrared beam responsible for measuring accommodation is reflected by the hot mirror into the eye and returns via the same path. The mechanics of the set-up facilitated the presentation of target vergences of −1.00, −2.00 and −3.00 D to stimulate accommodation. Hence, the accommodative demands were 1.00, 2.00, and 3.00 D. The target vergence was changed manually between trials. Subjects were instructed to focus on the centre of the target and to ‘keep it clear’ at all times, i.e. as sharp as possible (Stark and Atchison 1994). For each subject, 20 s of accommodation data was recorded at each of the three accommodative demands. The order of testing was randomised.

## Analysis

### Blink Removal

Any changes in accommodation of $\ge$0.45 D between consecutive readings were deleted. This equates to a change in accommodation velocity of ${>}10~\hbox {D}/\hbox {s}$, which is believed to be the maximum accommodation velocity (Campbell and Westheimer 1960). The deleted data were replaced by the average accommodation reading of the 300 msec prior to the blink.

**Fig. 2:**
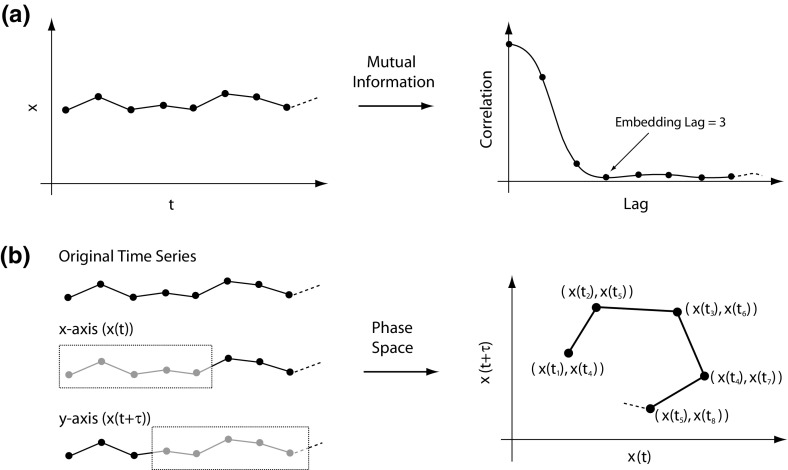
Principle of obtaining a multi-dimensional phase space plot from a one-dimensional time series. **a** The embedding lag is determined from the first minimum of the mutual information, which is three in the example given. **b** The original time series and the portion of the signal used for each of the two axes to obtain the phase space plot

### Chaos Theory Analysis

A marker of chaos, i.e. sensitive dependence on initial conditions, is a positive largest Lyapunov exponent (LE) (Rosenstein et al. 1993). The LE describes how the distance between nearby trajectories in phase space changes exponentially over time. All processing was carried out using custom written code in MATLAB (MathWorks, Massachusetts, USA). Prior to the calculation of any parameter associated with chaos theory analysis, the linear trend in the data was removed (Williams 1997).

#### Phase Space Reconstruction

Phase space is effectively a plot in which each axis represents a variable. When recording a single variable over time, as is commonly the case, and is the case here, a multi-dimensional plot is formed using lagged phase space (Liu 2010). Essentially, the time course signal is broken down into overlapping segments separated by an embedding lag $\tau$. Each segment is a ‘sub-series’ representing the values for that axis. The number of axis is governed by the embedding dimension *d*. The resulting phase space plot represents the dynamics of the system as it would be had each of the variables been known or measured. Using this method, a point, *p*(*t*), in a phase space with *d* dimensions is given by

1 $$\begin{aligned} \mathbf{p}(t)=\left[ x(t),x(t+\tau ),\ldots ,x(t+(d-1)\tau )\right] , \end{aligned}$$

where the embedding lag $\tau = t_{0}+ i\Delta t$, with $\Delta t$ being the time between frames (Liu 2010). The number of data points, $n_{\mathrm{ss}}$, per sub-series is given by

2 $$\begin{aligned} n_{\mathrm{ss}} =N-(d-1)\times i, \end{aligned}$$

where *N* is the total number of data points in the original time record and *i* is the embedding lag in units of data points.

**Fig. 3:**
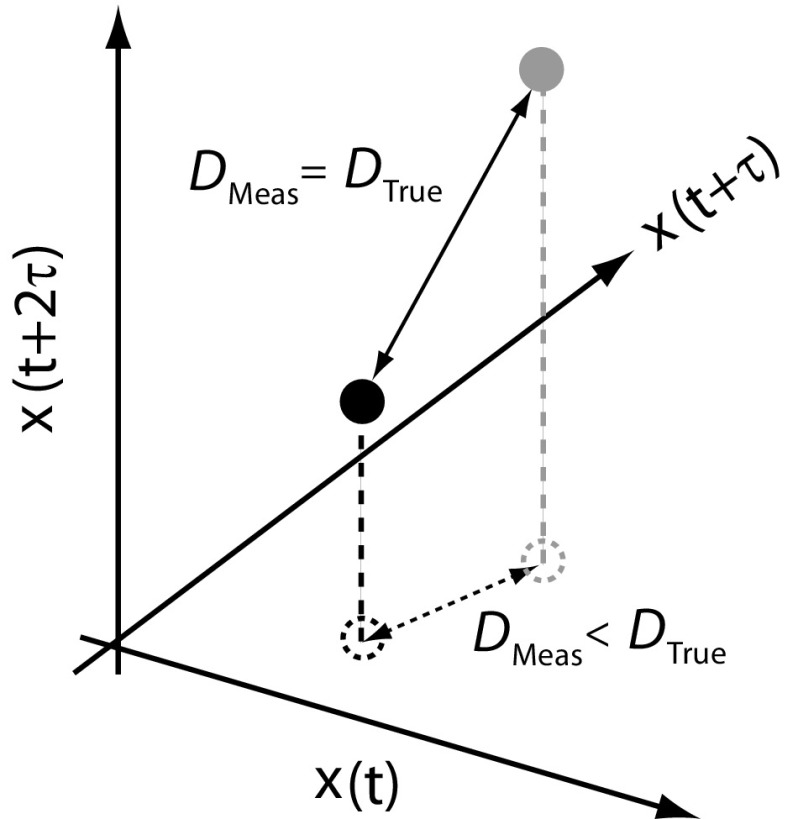
Principle of obtaining the correct embedding dimension. The correct dimension is three in the example shown. If the two points are embedded in too low a dimension, two in the example, the points appear artificially close

We determined the embedding lag using the first minimum of the mutual information (Williams 1997). As illustrated in Fig. 2a, the mutual information is in effect a correlation between the signal and a delayed version of itself. A schematic of obtaining a multi-dimensional phase space plot based on the determined lag is shown in Fig. 2b. In the example shown, the embedding dimension is two. For two time series *x* and *y*, the mutual information is given by

3 $$\begin{aligned} I_{XY} =\sum _{x_i ,y_j} {P_{XY} (x_i ,y_j )\log _2 \left( {\frac{P_{XY} (x_i ,y_j )}{P_X (x_i )P_Y (y_j )}} \right) } , \end{aligned}$$

where $P_{XY}$ is the joint probability, and $P_{X}$ and $P_{Y}$ are the marginal probabilities (Williams 1997). To determine $\tau$, the two time series are the original time series and the lagged time series. Hence,

4 $$\begin{aligned} I(\tau )=\sum _{x(t),x(t+\tau )} {P(x(t),x(t+\tau ))\log _2 \left( {\frac{P(x(t),x(t+\tau ))}{P(x(t))P(x(t+\tau ))}} \right) } . \end{aligned}$$

$I(\tau$) was calculated for each accommodation record for a lag of $i = 1{-}50$ data points.

To determine the correct dimension, the phase space plot is obtained for a number of dimensions. The separation of neighbouring points is then calculated for each dimension. If the dimension is too low, the points are artificially too close and referred to as false nearest neighbours (FNNs) (Kennel et al. 1992). The correct dimension is the one in which further increases in the dimension do not change the distance between the majority of data points and so the number of FNNs falls below a given threshold. An illustration of the effect of an incorrect and correct dimension is shown in Fig. 3. A data point located in *d*-dimensional space is given by

5 $$\begin{aligned} \mathbf{p}(t)=\left[ x(t),x(t+\tau ),\ldots ,x(t+(d-1)\tau )\right] , \end{aligned}$$

and its nearest neighbour is given by

6 $$\begin{aligned} \mathbf{p}_{\mathbf{NN}} (t_{\mathrm{NN}} )=\left[ x(t_{\mathrm{NN}} ),x(t_{\mathrm{NN}} +\tau ),\ldots ,x(t_{\mathrm{NN}} +(d-1)\tau )\right] . \end{aligned}$$

The distance between the points is

7 $$\begin{aligned} R_d^2 (\mathbf{p},\mathbf{p}_{\mathbf{NN}} )=\sum _{k=1}^d \left[ x(t +(k-1)\tau )-x(t_{\mathrm{NN}} +(k-1)\tau )\right] ^{2}. \end{aligned}$$

The separation of points when the dimension increases by one is given by

8 $$\begin{aligned} R_{d+1}^2 (\mathbf{p},\mathbf{p}_{\mathbf{NN}} )=R_d^2 (\mathbf{p},\mathbf{p}_{\mathbf{NN}} )+\left[ {x(t+\hbox {d}\tau )-x(t_{\mathrm{NN}} +\hbox {d}\tau )} \right] ^{2}. \end{aligned}$$

Hence, the change in distance can be calculated as

9 $$\begin{aligned} \sqrt{\frac{R_{d+1}^2 (\mathbf{p},\mathbf{p}_{\mathbf{NN}} )-R_d^2 (\mathbf{p},\mathbf{p}_{\mathbf{NN}} )}{R_d^2 (\mathbf{p},\mathbf{p}_{\mathbf{NN}} )}}=\frac{\left| {x(t+\hbox {d}\tau )-x(t_{\mathrm{NN}} +\hbox {d}\tau )} \right| }{R_d (\mathbf{p},\mathbf{p}_{\mathbf{NN}} )}. \end{aligned}$$

A point is considered a FNN if two conditions are satisfied:

10 $$\begin{aligned} \sqrt{\frac{R_{d+1}^2 (\mathbf{p},\mathbf{p}_{\mathbf{NN}} )-R_d^2 (\mathbf{p},\mathbf{p}_{\mathbf{NN}} )}{R_d^2 (\mathbf{p},\mathbf{p}_{\mathbf{NN}} )}}>R_{\mathrm{Tol}} , \end{aligned}$$

and

11 $$\begin{aligned} \frac{R_{d+1}}{R_A} >A_{\mathrm{Tol}}. \end{aligned}$$

where $R_{A}$ is the standard deviation of the time series (Kennel et al. 1992). Condition two prevents the number of FNNs rising again as *d* increases beyond the appropriate dimension, owing to the fact that the nearest neighbour of a point may not necessarily be the one closest to it. For a given dimension, the nearest neighbour of each point is determined and Eqs. (10) and (11) are evaluated for each pair of points. The correct embedding dimension is the value in which the number of FNNs falls below a given threshold.

For each accommodation record, the percentage of FNNs was determined for $d = 1:10.~R_{\mathrm{Tol}}$ was set to 15 (Su et al. 2008) and $A_{\mathrm{Tol}}$ was set to 5. The embedding dimension was taken as the dimension in which the percentage of FNNs was $\le$5%.

#### Lyapunov Exponent Calculation

**Fig. 4:**
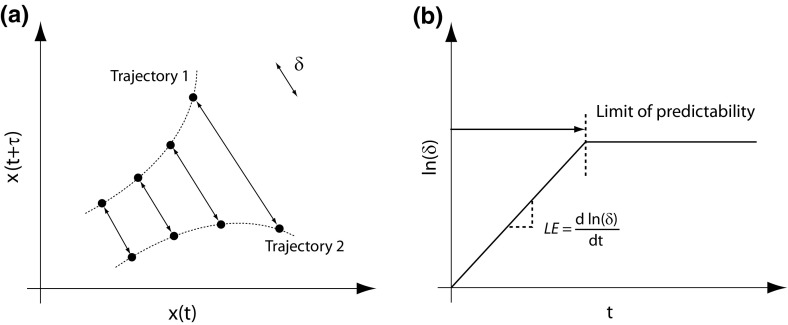
Determination of the Lyapunov exponent for a series with an embedding dimension of two. **a** Schematic of the divergence of nearby trajectories in phase space. **b** How the LE is obtained from the separation of neighbouring trajectories over time. The LE is the slope of the natural logarithm of the divergence of neighbouring trajectories over time. For a chaotic time series there is an initial rise in the natural logarithm of divergence over time and so the LE is positive

The LE represents the rate of exponential divergence (separation) of nearby trajectories in phase space. Figure 4a shows a schematic example of the phase space plot for a chaotic time series with an embedding dimension of two. The time evolution of the separation ($\delta$) of two nearby trajectories is also shown. Figure 4b shows the plot of the natural logarithm of the divergences versus time for the series. For a chaotic time series, there is an initial rise in the divergence of nearby trajectories. The slope of the linear rise is the LE. The end of the linear rise is the limit of predictability. Beyond this time the trajectories can be considered as effectively moving randomly with respect to each other. For a time series consisting of noise there is no relationship between the trajectories and so the plot is a continuous horizontal line ($\hbox {LE} = 0$). A periodic signal also has a value of zero.

To calculate the LE we used the algorithm of Rosenstein et al. (1993). This algorithm is robust to noise and also suitable for small data sets. The divergence of trajectories at a time *t* is given by

12 $$\begin{aligned} d(t)=Ce^{\mathrm{Lt}}, \end{aligned}$$

where *C* is a constant and *L* is the largest Lyapunov exponent. The nearest neighbour is found for each data point in the reconstructed phase space. In order to consider the two points to represent two different nearby trajectories the neighbour has to be separated in time by more than the average period of the time series. This is calculated as the inverse of the mean frequency of the power spectrum (Rosenstein et al. 1993). From Eq. (12), for the *j*th pair of neighbours, and time step *i*, the separation is

13 $$\begin{aligned} d_j (i)=Ce^{\mathrm{L}(i\Delta t)}, \end{aligned}$$

where $\Delta t$ is the time between frames. Hence

14 $$\begin{aligned} \ln d_j (i)=\ln C+L(i\Delta t). \end{aligned}$$

For each accommodation record, the left-hand side of Eq. (14) was evaluated for $i = 1:100$. From this, a plot of the natural logarithm of divergence, averaged across neighbours, versus time $(i\Delta t$) was obtained (Rosenstein et al. 1993). For plots that showed evidence of an initial linear rise, the data were fitted using two straight lines given by

15 $$\begin{aligned} y_1 =m_1 x(1,2,\ldots ,n)+C_1 , \end{aligned}$$

and

16 $$\begin{aligned} y_2 =m_2 x(n,n+1,\ldots ,100)+C_2 . \end{aligned}$$

*n* is the break-point time step, determined as the value which minimises

17 $$\begin{aligned} m_2 x(n)+C_2 -(m_1 x(n)+C_1 ). \end{aligned}$$

Hence $m_{2}$ was determined as the LE, and $(n\Delta t$) the limit of predictability.

#### Transitivity

To confirm chaos, we also tested the accommodation records for topological transitivity using the recurrence plot and the method proposed by Hirata and Aihara (2010). A recurrence plot is an image of a matrix consisting of ones and zeros. For an index (*i*, *j*), which represent the time indices, if the separation of two points in phase space is within a given threshold $\varepsilon$, the value is one. Otherwise the value is zero. Hence, effectively a recurrence plot is a plot of all the pairs of times where the trajectory revisits the same point in phase space. Hirata and Aihara have shown that if the minimum, of the maximum plotted row for each column, is greater than the maximum of the minimum plotted row for each column, then the phase space is topologically transitive. The bottom left-hand corner of the matrix represents (0,0).

There are a number of ways in which the threshold can be chosen for a recurrence plot. One is to set the threshold to a value that gives a fixed density (recurrence rate) of plotted points (Hirata and Aihara 2010). When testing for transitivity, too high a threshold value can result in false positives, whereas too low a threshold can result in false negatives. Hirata and Aihara have shown that the appropriate threshold depends on factors such as the noise level in the signal and the length of the signal. As this is the first time transitivity of the accommodation microfluctuations has been studied, the threshold value for the recurrence plot was chosen such that the recurrence rate was 50%.

### Traditional Analysis

For comparison purposes the power spectrum was calculated for each measurement record using the *periodogram* function in MATLAB, which is part of the MATLAB signal processing toolbox. This calculates the power spectral density of a time series. A Hanning window was used to reduce spectral leakage. Again the data were linearly detrended as failure to do so results in artificially high power spectral densities at low frequencies (Bendat and Piersol 2000). The area under the LFC (${<}0.6~\hbox {Hz}$) and high-frequency component (HFC) (1–2.3 Hz) were calculated. Prior to detrending, the standard deviation of the fluctuations and the mean accommodation level were also calculated for each record.

### Statistical Analysis

All statistical analysis was carried out using SPSS (v.21, IBM). Bonferroni post hoc testing was used in which the *p* value is adjusted for multiple comparisons. For example, when comparing the microfluctuations across refractive groups, there are three comparisons: EMM versus EOM, EMM versus LOM, and EOM versus LOM. In this case, rather than a statistically significant difference being represented by $p<0.05$, a value of $p<0.05/3$ is used.

## Results

### Chaos Theory Analysis

**Fig. 5:**
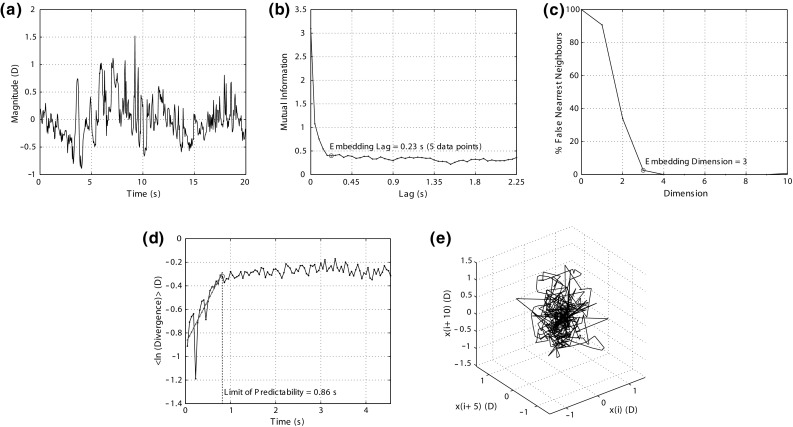
Chaos theory results for each processing step for an LOM at a 2 D accommodative demand. **a** Detrended accommodation time trace. **b** Mutual information. The first minimum is the embedding lag which is five data points. **c** The percentage of false nearest neighbours versus embedding dimension. The dimension is three in this case as this results in $\le$5% false nearest neighbours. **d** The time evolution of the average separation of nearby trajectories in phase space. The slope of the linear rise is the LE. The end of the linear rise is the limit of predictability. **e** The reconstructed phase space

**Fig. 6:**
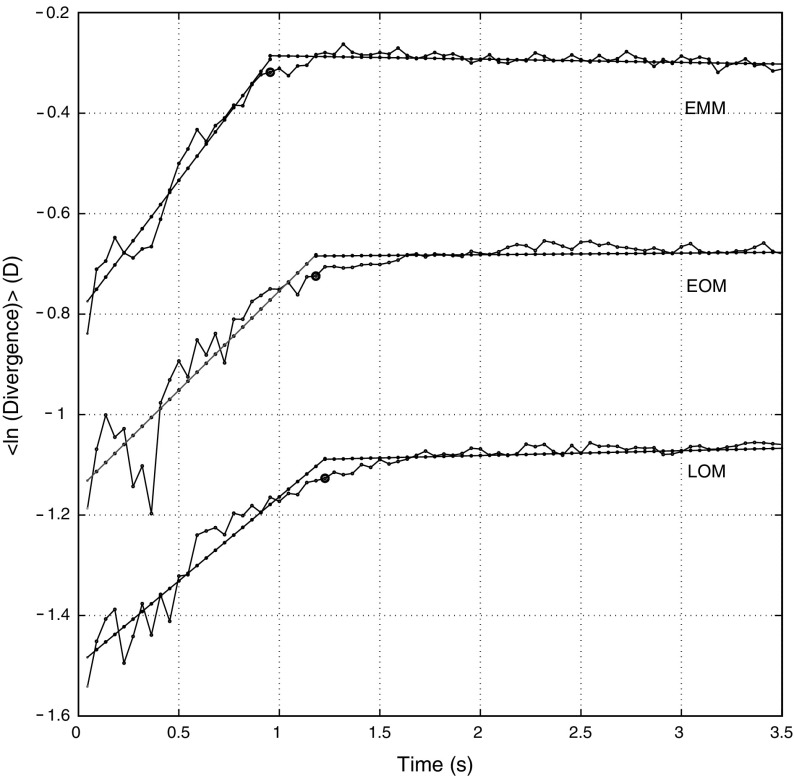
Divergence plots for each refractive group averaged across all accommodative demands. Also shown is the fit to each *curve*. The gradient of the linear rise is the LE. *Circles* indicate the limit of predictability

Figure 5 shows an example of the chaos theory results for each processing step for an LOM and 2 D accommodative demand. A mixed ANOVA looking at demand as “within-subjects effect”, with refractive error group as “between-subjects effect”, was calculated for the LE, embedding lag, embedding dimension, and limit of predictability. The ANOVA tests revealed insufficient evidence that accommodative demand had an effect ($p > 0.05$) for the LE, embedding lag, embedding dimension, and limit of predictability. The analysis showed a statistically significant effect of refractive error on both the LE ($p = 0.038, F = 4.084$) and embedding dimension ($p = 0.014, F = 5.735$). Post hoc analysis revealed that the EMMs had a higher LE than the LOMs, $0.64\pm 0.33$ versus $0.39\pm 0.20~\hbox {D}/\hbox {s}$, ($p = 0.015$), but there was insufficient evidence of a difference between EMMs and EOMs $(p > 0.05/3)$ or EOMs and LOMs $(p > 0.05/3).$ In addition, the EMMs had a lower embedding dimension than the LOMs, $3.28\pm 0.46$ versus $3.67\pm 0.49~\hbox {D}/\hbox {s}$, ($p = 0.005$). Again there was insufficient evidence of a difference between EMMs and EOMs or EOMs and LOMs. Figure 6 shows the divergence plots averaged across accommodative demand for each refractive group. The mean values for each parameter across refractive error and accommodative demand were: LE, $0.50\pm 0.29~\hbox {D}/\hbox {s}$; embedding lag, $0.30\pm 0.10~\hbox {s}$; embedding dimension, 3 $(3.44 \pm 0.50$); predictability $1.21\pm 0.57~\hbox {s}$. The results for each refractive group averaged across accommodative demand are shown in Table 1. There was insufficient evidence of a correlation between the average LE of each subject (i.e. averaged across accommodative demand) and SER ($p = 0.09$).

**Fig. 7:**
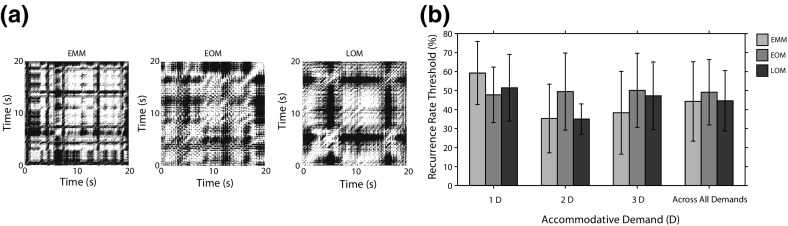
**a** Recurrence plots for three subjects for a 3 D accommodative demand. *White* represents a value of one. **b** Plot of the minimum recurrence rate for topological transitivity to test positive

Figure 7a shows examples of the recurrence plot for an EMM, EOM and LOM for a 3 D accommodative demand. Although some similarities were evident, in general we found that each data record had its own unique recurrence plot. Topological transitivity was confirmed in 37 out of 54 accommodation records (three demands for each of the 18 subjects). We investigated the effect of the recurrence plot density on whether topological transitivity is detected. Figure 7b shows the minimum recurrence rate needed for a record to test positive for topological transitivity. The plot shows the average value for each refractive group for each demand and for each refractive group across all demands. A mixed ANOVA revealed that the minimum recurrence rate was affected by demand ($p=0.035,F=3.754$). Post hoc analysis revealed that the threshold was higher for 1 D than for 2 D ($p = 0.012$), $52.8\pm 16.1$ versus $39.9\pm 16.8\%$. We found insufficient evidence of an effect of refractive error.

**Table 1:** Chaos parameters for each group averaged across accommodative demand

Parameter	Refractive group			
	EMM	EOM	LOM	All
Em. lag (s)	$0.29 \pm 0.11$	$0.31 \pm 0.07$	$0.32 \pm 0.13$	$0.30 \pm 0.10$
Em. dim.	$3.28 \pm 0.46^{\mathrm{a}}$	$3.39 \pm 0.50$	$3.67 \pm 0.49^{\mathrm{a}}$	$3.44 \pm 0.50$
Predict. (s)	$0.96 \pm 0.32$	$1.21 \pm 0.43$	$1.46 \pm 0.78$	$1.21 \pm 0.57$
LE (D/s)	$0.64 \pm 0.33^{\mathrm{a}}$	$0.46 \pm 0.28$	$0.39 \pm 0.20^{\mathrm{a}}$	$0.50 \pm 0.29$

$^{\mathrm{a}}$ Indicates a significant difference between groups $(p < 0.05/3)$

**Fig. 8:**
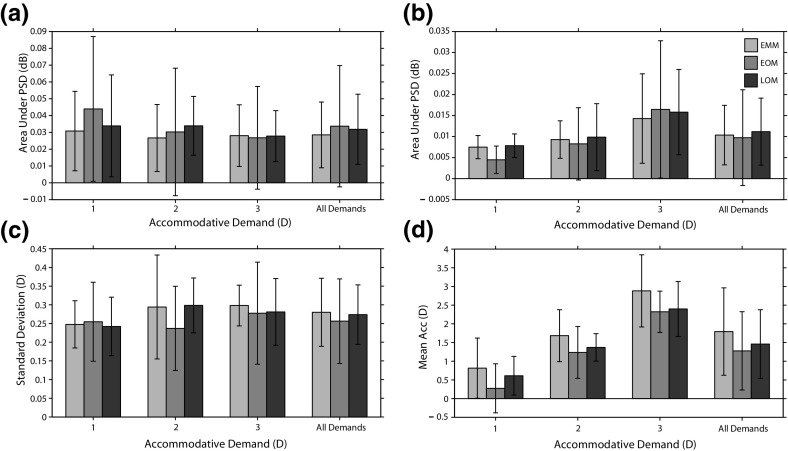
Results from the traditional analysis methods used to study changes in microfluctuations in accommodation. **a** Area under the LFC. **b** Area under the HFC. **c** Standard deviation. **d** Mean accommodation level

**Fig. 9:**
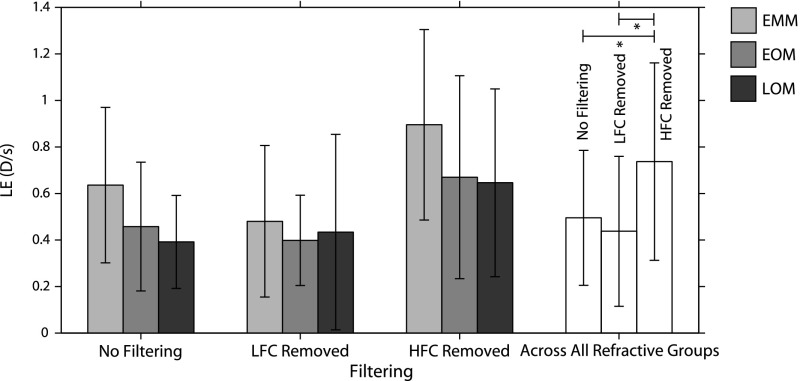
Effect of filtering on the LE. *Asterisk* represents statistically significant difference ($p < 0.05/3$)

### Traditional Analysis

Figure 8 shows the area under the LFC, area under the HFC, standard deviation, and mean accommodation level, for each refractive group for each demand and for each refractive group across all demands. A mixed ANOVA found insufficient evidence of refractive error having an effect on the area under the LFC, the area under the HFC, standard deviation of the fluctuations, and mean accommodation level ($p > 0.05$ in all cases). There was an effect of demand ($p = 0.012, F = 6.241$), for the area under the HFC only. Post hoc analysis revealed that the area under the HFC was significantly lower for 1 D in comparison to 2 D $(p = 0.009$). We found insufficient evidence of a correlation between the LE and the area under the LFC $(p = 0.53$), LE and the area under the HFC $(p = 0.06$), LE and mean accommodation level $(p = 0.09$), or LE and standard deviation $(p = 0.08$).

In order to determine the contribution of the LFC and HFC to the LE, we used a Butterworth filter in MATLAB to remove either the LFC or HFC from the accommodation record prior to calculating the LE. A Butterworth filter was chosen owing to its constant amplitude across the frequencies of interest that the filter passes. Figure 9 shows the effect of filtering on the LE for each refractive group for each filter and for each filter across all refractive groups. A mixed ANOVA revealed an effect of filtering $(p = 0.002, F = 10.039$) and refractive error $(p = 0.045, F = 3.842$), but not accommodative demand $(p = 0.951, F = 0.050$). There was also an interaction between demand and refractive error $(p = 0.018, F = 3.539$). Post hoc analysis revealed that filtering out the HFC resulted in a larger LE $(0.74 \pm 0.42~\hbox {D}/\hbox {s}$) than both the unfiltered data $[0.50 \pm 0.29~\hbox {D}/\hbox {s}~(p = 0.002)]$ and the data with the LFC filtered out $[0.44 \pm 0.32~\hbox {D}/\hbox {s}~(p=0.005)]$. There was insufficient evidence of an effect of refractive error under post-hoc testing.

## Discussion

In this paper, we determined the effect of refractive error and accommodative demand on chaos in microfluctuations in accommodation. For comparison purposes, we also used power spectrum analysis to ascertain any differences between the two methods of analysis.

### Chaotic Nature of Microfluctuations in Accommodation

The mean LE for each refractive group and accommodation demand was positive. We also found that in the majority of cases, the accommodation records tested positive for topological transitivity. Taken together, these results indicate that the microfluctuations in accommodation are chaotic. Hence, there are underlying laws governing the dynamics of the fluctuations. It was found that the EMMs had a statistically significant higher LE and lower embedding dimension than LOMs. There was insufficient evidence $(p > 0.05$) of a difference between refractive groups for the embedding lag and limit of predictability. An effect of accommodative demand was also not evident. As a reduction in the LE and increase in embedding dimension in systems such as the heart indicate stress or disease (Su et al. 2008; Yeragani et al. 2002; Rao and Yeragani 2001; Hampson et al. 2013), this suggests that the accommodation system of LOMs is under stress. It is interesting to note that although not statistically significant, the mean values of the chaos parameters for the EOMs fall between that of the EMMs and LOMs for the LE and embedding dimension, as well as the embedding lag and limit of predictability. Differences in the magnitude of the refractive error of the groups are not sufficient to explain the differences between the changes in chaos. For example, the EOMs had on average the biggest refractive error of the groups yet the LE fell between that of the EMMs and LOMs. Furthermore, we found insufficient evidence of a correlation between SER and the LE.

By increasing the recurrence rate beyond the chosen value of 50%, all accommodation records became topologically transient. When investigating the minimum recurrence rate necessary for a given accommodation record to test positive for topological transitivity, we found a statistically significant effect of demand, but not refractive error. The threshold recurrence rate for 2 D was lower than that of 1 D. This could reflect changes in the statistical properties of the accommodation system (Leahy et al. 2010), such as reduced noise at 2 D. Future work will involve investigating this further.

Advantages of chaos as a control strategy of physiological systems are that the system is able to easily adapt to changes in the environment. It may be that LOMs have a less adaptable accommodation system. The results from this study will aid in the development of models of accommodation microfluctuations and changes in myopia. For example, an embedding dimension of three indicates that there are of the order of three variables that control the microfluctuations in accommodation.

### Comparison with Previous Studies

The value of the LE and other phase space reconstruction parameters, i.e. embedding lag, embedding dimension, and predictability, found here are similar to those found in other studies which analysed microfluctuations in accommodation (Hampson and Mallen 2012, 2013; Sumida et al. 1994; Hampson et al. 2013; Iwase and Murata 1999). Although the LE and embedding dimension were found to be different for LOMs, using the traditional analysis methods of power spectrum analysis and the standard deviation did not reveal any refractive error differences. Day et al. for example found a significant effect of refractive error on the powers of the LFC and the standard deviation of the fluctuations. They found that the LFC power increases more rapidly with accommodative demand for EMMs and EOMs, in comparison to LOMs, and that the standard deviation of the fluctuations is higher in LOMs (Day et al. 2006).

One explanation for finding insufficient evidence of a difference in this study with regard to the LFC may be the way in which the data were processed. Here the linear trend was removed prior to processing the data. Failure to remove a linear trend results in an artificial increase in the power spectrum for low frequencies (Bendat and Piersol 2000). We reprocessed the power spectrum data without removing the linear trend and found that there was still insufficient evidence of an effect of refractive error, however. Interestingly, when the linear trend is not removed, we find the effect of demand becomes statistically significant for the area under the LFC as in other studies, e.g. Day et al. (2006). Another possible explanation may simply be that the subjects in this study did not have detectable significant differences in their power spectrum (or standard deviation). It has been shown that these measures vary greatly between subjects (Harb et al. 2006). Insufficient evidence of a difference in the power spectrum (and standard deviation) found here suggests chaos theory analysis is potentially a more sensitive marker of differences in accommodation function between refractive groups. This has been shown to be the case when determining the effect of adaptive optics correction of blur on microfluctuations in accommodation (Hampson et al. 2013) and also when studying other physiological signals such as the heartbeat (Yeragani et al. 2002).

### Potential Origin

A potential contributor to the chaotic dynamics is the heartbeat. The healthy heartbeat is itself chaotic (Sharma 2009), and several studies have shown that the HFC of the microfluctuations in accommodation is correlated with the heartbeat; see, for example, Winn et al. (1990), Collins et al. (1995), van der Heijde et al. (1996). Although the LFC region is considered to be part of the accommodation control system, part of this region is correlated with respiration and fluctuations in instantaneous pulse rate (Collins et al. 1995; van der Heijde et al. 1996). Both respiration and fluctuations in instantaneous pulse rate are chaotic (Poon and Merrill 1997; Su et al. 2008; Yeragani et al. 2002; Rao and Yeragani 2001; Donalson 1992). We found that filtering out the HFC increases the level of chaos in the accommodative microfluctuations and so the presence of the HFC potentially adversely affects the accommodation system. However, care must be taken when analysing a chaotic signal that has been filtered as it has been shown that this can distort the phase space plot and result in incorrect determination of the chaos theory parameters (Rosenstein and Collins 1994). Future work will include simultaneously measuring the pulse and the microfluctuations in accommodation in order to study further the connection between the heart and oculomotor system.

The level of chaos in heart rate variability has been found to depend mainly on the parasympathetic branch of the nervous system, with some effect due to the sympathetic branch (Hangerman et al. 1996). An increase and decrease in parasympathetic activity produce increases and decreases in the LE of heart rate variability, respectively. An increase and decrease in sympathetic activity have the opposite effect on the LE but to a lesser extent. During a task in which cognitive demand increased, Davies et al. (2005) have found that LOMs have increased sympathetic activity in the heart in comparison to EMMs. At first sight, this appears to be a possible explanation for the results found here. However, using a sympathomimetic and parasympathomimetic agent, Sumida et al. (1994) found an increase and decrease in the LE of microfluctuations in accommodation respectively. Further testing with more subjects is required to investigate this further. These studies suggest that the effects of the sympathetic and parasympathetic components of the nervous system controlling the heart, and those parts controlling the eye, potentially have different effects on the eye. Interestingly, atropine applied to the eye, which reduces stimulation of the parasympathetic nervous system, has been found to be effective in slowing myopia progression (Chia et al. 2012). Unfortunately, we do not have data on whether the myopic subjects used in this study were stable or progressing. Future work will include investigating the differences in chaos in the microfluctuations in accommodation in progressive versus stable myopes. Other studies that have investigated the accommodation system in stable and progressive myopes have found differences between these two groups. For example, Millodot (2015) found that the gradient of the stimulus response curve of accommodation is different.

Aside from the impact of heart rate variability and respiration on the LFC, part of the LFC is considered to be part of the accommodation control system owing to changes with depth of focus (Charman and Heron 1988, 2015; Winn 2000). Hence, this may be the mechanism which also imparts chaos in the microfluctuations in accommodation. The microfluctuations have also been found to be a potential cue in the accommodative step response (Metlapally et al. 2016). Future work will include using chaos theory to determine the effect of the LE on dynamics of the step response in different refractive groups (Seidel et al. 2005). It must be pointed out that the LE values found here are likely to be lower than those induced by fluctuations in the crystalline lens due to the tear film affecting the measured signal and reducing the chaos (Hampson and Mallen 2012; Jayakumar et al. 2013).

## Conclusion

In conclusion, we have found that the dynamics of the microfluctuations in accommodation remain chaotic irrespective of accommodative demand and refractive error. Chaos theory applied to microfluctuations in accommodation can reveal differences in EMMs compared to LOMs that are not detected using traditional analysis methods.

## References

[CR1] Bendat JS, Piersol AG (2000). Random data: analysis and measurement procedures.

[CR2] Campbell FW, Westheimer G (1960). Dynamics of accommodation responses of the human eye. J Physiol.

[CR3] Charman WN, Heron G (1988). Fluctuations in accommodation: a review. Ophthalmic Physiol Opt.

[CR4] Charman WN, Heron G (2015). Microfluctuations in accommodation: an update on their characteristics and possible role. Ophthalmic Physiol Opt.

[CR5] Chia A, Chua WH, Cheung YB, Wong WL, Lingham A, Fong A, Tan D (2012). Atropine for the treatment of childhood myopia: safety and efficacy of 0.5, 0.1 and 0.01% doses. Ophthalmology.

[CR6] Collins G (1937). The electronic refractometer. Br J Physiol Opt.

[CR7] Collins M, Davis B, Wood J (1995). Microfluctuations of steady-state accommodation and the cardiopulmonary system. Vis Res.

[CR8] Davies LN, Wolffsohn JS, Gilmartin B (2005). Cognition, ocular accommodation, and cardiovascular function in emmetropes and late-onset myopes. Invest Ophthalmol Vis Sci.

[CR9] Day M, Strang NC, Seidel D, Gray LS, Mallen EA (2006). Refractive group differences in accommodation microfluctuations with changing accommodation stimulus. Ophthalmic Physiol Opt.

[CR10] Donalson GC (1992). The chaotic behaviour of resting human respiration. Respir Physiol.

[CR11] Gambra E, Sawides L, Dorronsoro C, Marcos S (2009). Accommodative lag and fluctuations when optical aberrations are manipulated. J Vis.

[CR12] Gray LS, Winn B, Gilmartin B (1993). Accommodative microfluctuations and pupil diameter. Vis Res.

[CR13] Gray LS, Winn B, Gilmartin B (1993). Effect of target luminance on microfluctuations of accommodation. Ophthalmic Physiol Opt.

[CR14] Hampson KM, Cufflin MP, Mallen EA (2013). Effect of correction of aberration dynamics on chaos in human ocular accommodation. Opt Lett.

[CR15] Hampson KM, Mallen EA (2013). Correspondence of chaos in binocular aberration dynamics. Opt Lett.

[CR16] Hampson KM, Mallen EA (2012). Chaos in ocular aberration dynamics of the human eye. Biomed Opt Express.

[CR17] Hangerman I, Berglund M, Lorin M, Nowak J, Slyvén C (1996). Chaos-related deterministic regulation of heart rate variability in time-and frequency domains: effects of autonomic blockade and exercise. Cardiovasc Res.

[CR18] Harb E, Thorn F, Troilo D (2006). Characteristics of accommodative behavior during sustained reading in emmetropes and myopes. Vis Res.

[CR19] Hirata Y, Aihara K (2010). Devaney’s chaos on recurrence plots. Phys Rev E.

[CR20] Iwase H, Murata A (1999) Chaotic analysis of focal accommodation system and pupil area during VDT work. In: Proceedings of international conference, systems, man and cybernetics. pp 277–282

[CR21] Jayakumar V, Thapa D, Hutchings N, Lakshminarayanan V (2013). Are the fluctuations in dynamic anterior surface aberrations of the human eye chaotic?. Opt Lett.

[CR22] Kennel MB, Brown R, Abarbanel HD (1992). Determining embedding dimension for phase-space reconstruction using a geometrical construction. Phys Rev A.

[CR23] Leahy C, Leroux C, Dainty C, Diaz-Santana L (2010). Temporal dynamics and statistical characteristics of the microfluctuations of accommodation: dependence on the mean accommodative effort. Opt Express.

[CR24] Langaas T, Riddell PM, Svarverud E, Ystenaes AE, Langeggen I, Bruenech JR (2008). Variability of the accommodation response in early onset myopia. Optom Vis Sci.

[CR25] Langaas T, Riddell PM (2012). Accommodative instability: relationship to progression of early onset myopia. Clin Exp Optom.

[CR26] Liu Z (2010). Chaotic time series analysis. Math Probl Eng.

[CR27] McBrien NA, Millodot M (1986). The effect of refractive error on the accommodative response gradient. Ophthalmic Physiol Opt.

[CR28] Metlapally S, Tong JL, Tahir HJ, Schor CM (2016). Potential role for microfluctuations as a temporal directional cue to accommodation. J Vis.

[CR29] Millodot M (2015). The effect of refractive error on the accommodative response gradient: a summary and update. Ophthalmic Physiol Opt.

[CR30] Monticone PP, Menozzi MA (2011). A review on methods used to record and analyze microfluctuations of the accommodation in the human eye. J Eur Opt Soc Rapid Publ.

[CR31] Niwa K, Tokoro T (1998). Influence of spatial distribution with blur on fluctuations in accommodation. Optom Vis Sci.

[CR32] Poon CS, Merrill CK (1997). Decrease of cardiac chaos in congestive heart failure. Nature.

[CR33] Rao RK, Yeragani VK (2001). Decreased chaos and increased nonlinearity of heart rate time series in patients with panic disorder. Auton Neurosci.

[CR34] Rosenstein MT, Collins JJ, De Luca CJ (1993). A practical method for calculating largest Lyapunov exponents from small data sets. Physica D.

[CR35] Rosenstein MT, Collins JJ (1994). Visualizing the effects of filtering chaotic signals. Comput Graph.

[CR36] Seidel D, Gray LS, Heron G (2005). The effect of monocular and binocular viewing on the accommodation response to real targets in emmetropia and myopia. Optom Vis Sci.

[CR37] Sharma V (2009). Deterministic chaos and fractal complexity in the dynamics of cardiovascular behavior: perspectives on a new frontier. Open Cardiovasc Med J.

[CR38] Skinner JE (1994). Low-dimensional chaos in biological systems. Nat Biotechnol.

[CR39] Stark LR, Atchison DA (1997). Pupil size, mean accommodation response and the fluctuations of accommodation. Ophthalmic Physiol Opt.

[CR40] Stark LR, Atchison DA (1994). Subject instructions and methods of target presentation in accommodation research. Invest Ophthalmol Vis Sci.

[CR41] Su ZY, Wu T, Yang PH, Wang YT (2008). Dynamic analysis of heartbeat rate signals of epileptics using multidimensional phase space reconstruction approach. Phys A.

[CR42] Sumida T, Tahara T, Iwanaga H (1994). Physiological significance of the Shil’nikov phenomenon in the focal accommodation system of human eyes. Int J Bifurcat Chaos.

[CR43] van der Heijde GL, Beers AP, Dubbelman M (1996). Microfluctuations of steady-state accommodation measured with ultrasonography. Ophthalmic Physiol Opt.

[CR44] Williams GP (1997). Chaos theory tamed.

[CR45] Winn B, Franzén O, Richter H, Stark L (2000). Accommodative microflutuations: a mechanism for control of steady-state accommodation. Accommodation and vergence mechanisms in the visual system.

[CR46] Winn B, Pugh JR, Gilmartin B, Owens H (1990). Arterial pulse modulates steady-state ocular accommodation. Curr Eye Res.

[CR47] Wolffsohn JS, Gilmartin B, Mallen EA, Tsujimura S (2001). Continuous recording of accommodation and pupil size using the Shin-Nippon SRW-5000 autorefractor. Ophthalmic Physiol Opt.

[CR48] Yao P, Lin H, Huang J, Chu R, Jiang B (2010). Objective depth-of-focus is different from subjective depth-of-focus and correlated with accommodative microfluctuations. Vis Res.

[CR49] Yeragani VK, Rao KA, Smitha MR, Pohl RB, Balon R, Srinivasan K (2002). Diminished chaos of heart rate time series in patients with major depression. Biol Psychiatry.

